# Kaempferol improves acute kidney injury via inhibition of macrophage infiltration in septic mice

**DOI:** 10.1042/BSR20230873

**Published:** 2023-07-25

**Authors:** Zuqing Xu, Xiao Wang, Wenbin Kuang, Shiyang Wang, Yanli Zhao

**Affiliations:** 1 Department of Emergency Medicine Department, Shenzhen Longhua District Central Hospital, Shenzhen 518110, China; 2 Department of Medical Laboratory, Shenzhen Longhua District Central Hospital, Shenzhen 518110, China; 3 Department of Ultrasound, Shenzhen Longhua District Central Hospital, Shenzhen 518110, China; 4 Department of Laboratory, Shenzhen Longhua District Central Hospital, Shenzhen 518110, China

**Keywords:** Flavonoid, Immune cells, Inflammatory response, Macrophage recruitment, MCP-1

## Abstract

Sepsis contributes to life-threatening circulatory and organ dysfunction by dysregulating the host response to infection in critically ill patients. Treatment in an Intensive Care Unit (ICU) can improve the survival of patients who suffer from severe sepsis, but sepsis-associated acute kidney injury (SAKI) is still one of the main causes of death. The existing treatment is mainly focused on controlling microorganism induced infections by using drugs, such as ulinastatin and glucocorticoid. Also, it is well documented that kaempferol, a flavonoid derived from plant sources, improves septic mouse survival via anti-inflammatory response. However, the mechanism of anti-inflammatory response mediated by this flavonoid compound was little known. This study aims to demonstrate the mechanisms of inflammatory response regulated by kaempferol treatment during sepsis. We perform cecal ligation and puncture (CLP) injury as a sepsis mouse model and evaluate organ injury in sepsis. The molecular (qRT-PCR and Western Blot) and cellular profiling (IHC staining and Flow Cytometry) of the immune responses illustrates that kaempferol decreases the expression of adhesion molecular genes (ICAM-1 and VCAM-1) and monocyte chemoattractant protein-1 (MCP-1), thereby inhibiting F4/80+ macrophages infiltration in CLP-induced acute kidney injury. Our data suggested that kaempferol alleviates acute kidney injury via regulating F4/80+ macrophages infiltration in CLP-induced acute kidney injury.

## Introduction

Sepsis induced immune dysfunction, which contributed to an immune imbalance between the host immune response and microorganism infection and subsequently contributed to severe organ dysfunction. The incidence rate and mortality of septic shock rose gradually in the world, and 40–50% of septic shock caused an immune storm that primarily contributes to multiple organ injury, especially acute kidney injury [1,2]. Moreover, renal dysfunction, prolonged intensive care unit treated days (ICUs administration), has diminished the survival rate of patients suffered from acute kidney injury [3]. Although the administration of modern antibiotics and respiratory resuscitation could be useful to reduce the mortality of sepsis in critically ill patients, the molecular and cellular mechanism of acute renal injury are still unclear during sepsis [4,5] The possible pathogenic mechanisms include microcirculation dysfunction, cell metabolism disorder, renal inflammatory infiltration and oxidative stress injury caused by changes in renal hemodynamics [6]. Mounting studies demonstrated that pro-inflammatory responses accelerated platelets activation, immune cells interaction, and cytokine storm during the development of sepsis [7,8]. Also, some novel therapeutic targets play a vital role in sepsis therapy, such as CD39 [9], glycocalyx degradation [10], immune cell apoptosis [11], cytokine antagonists [12], and cytokines treatment [13]. Thus, the balance between pro-inflammatory and anti-inflammatory response is a central mediator for diminishing the mortality of septic shock in clinical administration.

Kaempferol, a flavonoid derived from plant and plant-based foods [14], could improve parkinson's disease [15], atherosclerosis [16], obesity [17], inflammatory bowel disease [18], and cancers [19]. In the context of inflammatory response, kaempferol could regulate the inflammatory response by controlling CD3+ T cell infiltration [20], CD4+FoxP3+ regulatory T cells [21], and Ag-specific T cells in both *in vivo* and *in vitro* experiments [20–22]. Moreover, kaempferol inhibited inflammatory markers such as Cyclooxygenase-2 (COX-2), tumor necrosis factor-α (TNF-α), Interleukin-1β (IL-1β), Interleukin-6 (IL-6), Intercellular cell adhesion molecule-1 (ICAM-1), and vascular cell adhesion molecule-1 (VCAM-1) [23,24].

Macrophage is a master regulator of the inflammatory response, regulating cardiovascular disease, heart transplantation, tissue regeneration and repair, as well as obesity [25,26]. Signaling pathways associated with macrophages, including MCP-1/CCR-2, PGC-1-SPP1, ANG/TIE2, DLL1-NOTCH, and NF-κB, were responsible for chemotaxis, cell recruitment, and angiogenesis in tissue injury and regeneration [27,28]. More interestingly, Kaempferol treatment decreased the production of pro-inflammatory cytokines (TNF-α and IL-1β) and inhibited apoptosis in LPS-induced HK-2 cells via regulating NF-κB/AKT signaling [29]. Kaempferol also improved cisplatin-induced nephrotoxicity by reducing inflammation, apoptosis and oxidative stress via NF-κB and ERK pathways [30]. In another study, kaempferol treatment reduced the protein levels of p-ERK, p38 and p-JNK, which inhibited MAPKs signaling pathway in LPS-induced lung injury [31]. Although kaempferol alleviated inflammation, there were little relevant data whether kaempferol could alleviated sepsis-associated acute kidney injury (SAKI) through mediating macrophages.

In the present study, we used cecal ligation and puncture (CLP) injury as a sepsis mouse model to identify the inflammatory mechanism of kaempferol treatment improved SAKI. Our data demonstrated that kaempferol alleviated SAKI at 24 h and inhibited significant splenomegaly at 48 h during sepsis. Moreover, kaempferol alleviated kidney damage. Molecular and cellular profiling of the inflammatory responses illustrated that kaempferol inhibited the expression of adhesion molecular genes (ICAM-1 and VCAM-1) and monocyte chemoattractant protein-1 (MCP-1), declining the number of F480+ macrophages infiltration in CLP-induced kidney injury.

## Materials and methods

### Clinical samples

All clinical patients’ serum was performed under protocols approved by the Ethics Review Committee of Shenzhen Longhua District Central Hospital in accordance with World Medical Association Declaration of Helsinki (WMA) (Ethical Application Ref: AF/SC-08/01.1/2021-135-01) and all written informed consent of the subjects have been included in the Ethics approval.

### Animal

All animals were approved by the Ethics Review Committee of Guangdong Medical University in accordance with the principles of animal welfare in the Institutional Animal Care (Ethical Application Ref: GDY2102490). Adult male ICR (Age: 6–8w) were procured from Guangdong Medical Laboratory Animal Center (Foshan, Guangdong, China) and all animal experiments took place at department of medical laboratory, Shenzhen Longhua District Central Hospital (Affiliated Central Hospital of Shenzhen Longhua District, Guangdong Medical University). The mice were given rodent chow (SPF Grade, Guangdong Medical Laboratory Animal Center, Foshan, Guangdong, China) and water ad libitum. The temperature and humidity were maintained at 25 ± 2°C and 55 ± 10%, respectively, with 12-h light/dark cycle. The kaempferol were purchased from Meilunbio Co., Ltd. (MB6888, Meilun, Dalian, China). The optimized concentration (1 mg/kg) of kaempferol has been demonstrated in CLP mouse model, so kaempferol (1 mg/kg) was diluted in saline before administration [32]. Adult male ICR were randomly divided into three groups, sham group (*n*=5), saline-treated CLP group (*n*=12), and kaempferol (1 mg/kg)-treated CLP group (*n*=12). The intragastricinjection of kaempferol was administrated once after CLP injury, and mice were killed at 24 and 48 h after CLP injury, respectively. All animals were killed after anesthetizing with 2% isofluorane in 95% oxygen and analyzed in a blinded manner.

### Animal sepsis model-cecal ligation and puncture injury (CLP)

The cecal ligation and puncture injury was performed as described in a previous study [33]. Briefly, adult ICR mice were anesthetized and maintained with 2% isofluorane in 95% oxygen until finishing CLP surgery. Before surgery, we confirm the animal’s anesthesia status by examining the mouse’s response to the pain stimuli [34]. After completing the examination, the abdominal skin and muscle were incised, and the cecum was exposed and punctured with a suitable needle after cecum ligation. When CLP surgery was finished, the muscles of the abdomen and skin were sewn up with medical sutures and the mice were placed under warming light. At the endpoint of experiment (24 and 48 h), animals were anesthetized and maintained with 2% isofluorane in 95% oxygen and then sacrificed by cervical dislocation.

### Quantitative real-time PCR (qRT-PCR)

For cDNA synthesis, total RNA was reverse transcribed to cDNA with Transcript First-Strand cDNA Synthesis SuperMix (Takara Bio Inc, Dalian, China) according to the instructions. For qPCR, SYBR Premix Ex Taq kit (Takara Bio Inc, Dalian, Liaoning, China) was used. Real-time PCR was performed with ABI 7500 Real-Time PCR System. Gene expression were calculated using the 2^−ΔΔCt^ method and normalized to GAPDH. All the primers of qRT-PCR are listed in Supplementary Table S1.

### Western blot

Kidney tissues were harvested after washing with ice-cold PBS and lysed for 30 min in ice-cold RIPA lysis buffer containing protease inhibitor cocktail (Roche, Indianapolis, IN, U.S.A.) and separated by 10% SDS-PAGE. After transferring protein on to polyvinylidene fluoride (PVDF) membranes (Millipore, Billerica, MA, U.S.A.), the PVDF membranes were incubated overnight at 4°C with primary antibodies: VCAM-1 (CST, 39036S#), ICAM-1 (Novus, NBP2-22541), MCP-1 (Novus, NBP2-22115SS), and β-tubulin (CST, #2128). The protein expression levels of specific gene were calculated as the relative band density to that of β-tubulin using ImageJ software (National Institute of Health, Bethesda, MD, U.S.A.).

### Immunohistochemistry and H&E staining

Mouse kidneys were perfused and fixed in 4% paraformaldehyde at 4°C overnight. The fixed kidneys were dehydrated in a Dehydrator (Donatello, DIAPATH, Italy) and embedded in an Embedding machine (JB-P5, Wuhan Junjie Electronics Co., Ltd, Wuhan, China) and then stained with H&E staining kit (Maixin, Fuzhou, China), and immunohistochemistry (Servicebio, Wuhan, China) in accordance with the manufacturer’s instructions. The primary antibody of macrophage staining: F4/80 (CST, Cat#30325)

### Flow cytometry

Kidney tissues were sectioned into small fragments with sterile scissors and consequently dissociated with a mixed buffer of Collagenase Type II (Life Technologies, 17101015, Carlsbad, CA, U.S.A.) and Dispase (Solarbio, D6430-1g, Beijing, China) at 37°C for 30 min. After dissociation, the tissues were added with 10% FBS (Gibico, Carlsbad, CA, U.S.A.) to stop the enzymatic action and washed once with PBS. Red blood cells were removed with 1× Red lysis buffer for 2–3 min at room temperature (Solarbio, Beijing, China). The collected kidney tissues were staining with F4/80-APC (Biolegend, 123116, San Diego, U.S.A.). After incubation at 4°C for 30 min in the dark, the cells were washed with PBS, and then analyzed using a BD FACS Aria II flow cytometer (BD Biosciences, Franklin Lakes, NJ, U.S.A.).

### Statistical analysis

The results were presented as the mean values ± standard error of mean (mean ± S.E.M), and data were statistically performed using GraphPad Prism 8.0 software (NIH, U.S.A.). The two tailed Student’s *t-*test was used to calculate statistical differences between two groups, while One-way ANOVA followed by Turkey’s method for multiple comparisons was performed in more than two groups. *P<0.05, **P<0.01, and ***P<0.001 are statistically significant.

## Results

### The expression of pro-inflammatory markers and inflammatory cells in septic patients

**Figure 1:**
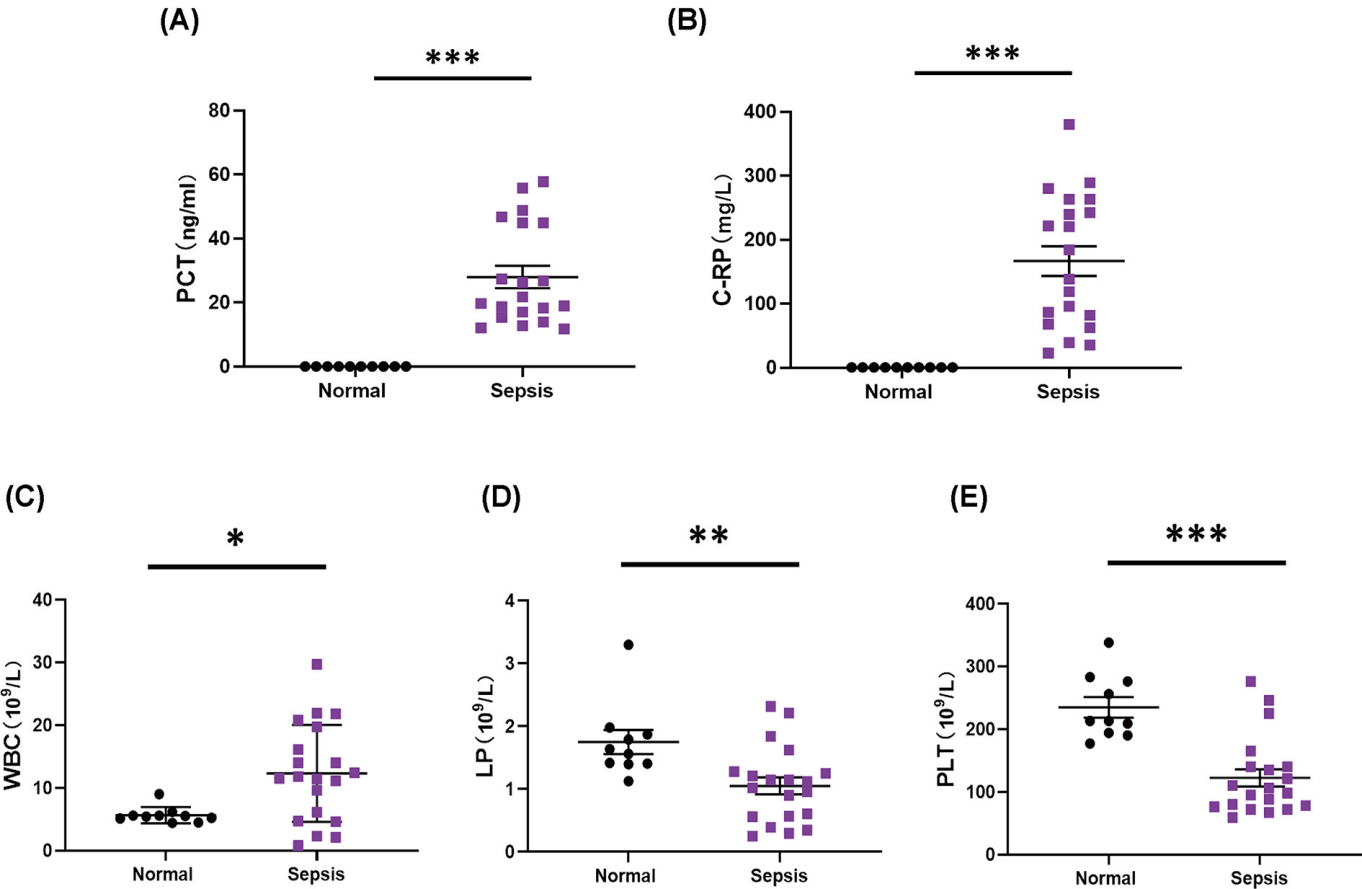
The expression of pro-inflammatory markers and inflammatory cells in septic patients The serum level of (**A**) procalcitonin, (**B**) C-reactive protein, (**C**) white blood cells, (**D**) lymphocytes, and (**E**) platelets in normal (*n*=10) and septic patients (*n*=20) (mean ± S.E.M, *P<0.05, **P<0.01, ***P<0.001).

Levels of inflammatory factors, such as procalcitonin (PCT) and C-reactive protein (C-RP), are key diagnostic indicators of clinical inflammation. Thus, we tested whether these two indicators were different in the blood of hospitalized septic patients in ICU and normal group. The results showed that the serum level of PCT in sepsis group was up-regulated significantly from 10 to 60 ng/ml compared with 0 ng/ml in normal (Figure 1A). In addition, serum C-RP levels (ranging from 20 to 400 mg/L) increased significantly to 40- to 800-fold compared with normal (less than 0.5 mg/L) in patients with sepsis (Figure 1B). These data suggested that PCT and CRP were observed to considerably increase in septic patients.

Leukocytes, lymphocytes, and platelets were responsible for protecting the body against infection, and the number of these immune cells would be varied in patients during severe sepsis. Our results showed the number of white blood cells (WBCs) changed from 3 to 30 (10^9^/L) in the sepsis group, while that of the normal group changed from 4 to 10 (10^9^/L). The data showed that the amount of WBC would increase remarkably during sepsis (Figure 1C). The average number of lymphocytes in the sepsis group was less than 1.1 (10^9^/L), while that of the normal group was roughly 1.6 (10^9^/L), suggesting the number of lymphocytes decreased notably during sepsis (Figure 1D). Furthermore, the average number of platelets in septic patients group concentrated at 120 (10^9^/L), showing a significant decrease of 100 (10^9^/L) compared with that of the normal group (230 × 10^9^/L) (Figure 1E).

### The effects of kaempferol on organ weight and acute kidney injury in SAKI

**Figure 2:**
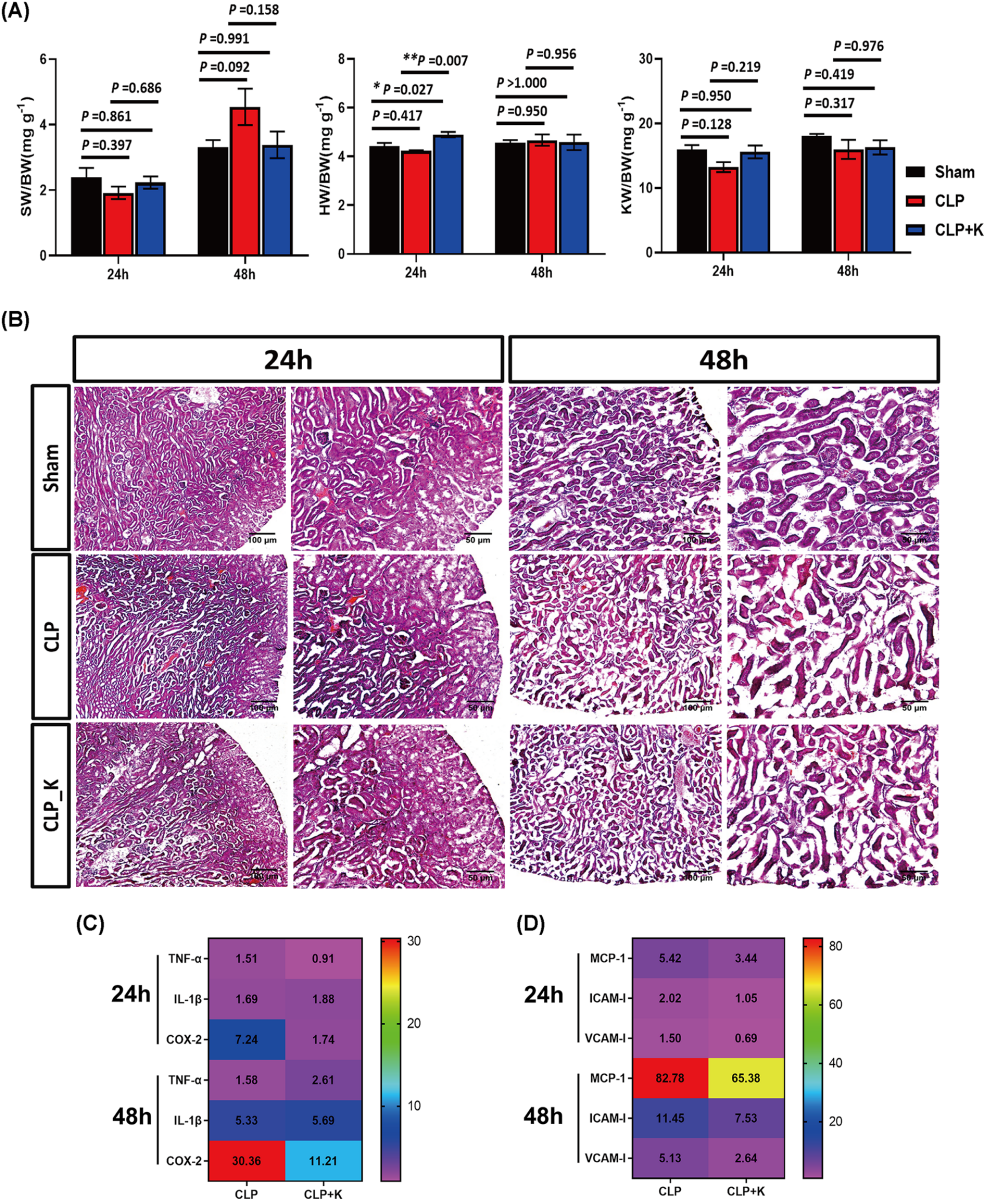
The effects of kaempferol on organ weight and acute kidney injury during sepsis The ratio of kidney, heart, spleen to body weight in sham, saline-treated CLP mice, and kaempferol treated CLP mice at (**A**) 24 and 48 h, and (**B**) the morphology of sham, saline-treated CLP mice, and kaempferol treated CLP mice at 24 and 48 h, (**C**) the mRNA expression level (TNF-α, IL-1β, and COX-2) of saline-treated CLP mice and kaempferol treated CLP mice at 24 and 48 h; (**D**) the mRNA expression level (MCP-1, VCAM-1, and ICAM-1) of saline-treated CLP mice and kaempferol treated CLP mice at 24 and 48 h (*n* = 3–5 per group, mean ± S.E.M, *P<0.05, **P<0.01, ***P<0.001).

It is well documented that sepsis leads to multiple organ function damage through inflammatory storms, especially kidney damage. Kaempferol (1 mg/kg) administration prolonged mice survival to approximately 20% during sepsis [32]. To evaluate the effect of kaempferol on CLP induced organ damage during sepsis, we identified the weight of organs in sham group, saline-treated CLP group, and kaempferol-treated CLP group at 24, and 48 h after CLP injury respectively. Our data demonstrated that the ratio of kidney/body (KW/BW) (*P*=0.128) and spleen/body (SW/BW) (*P*=0.397) reduced in CLP group compared with the sham group, and also there was no remarkable difference in the kaempferol-treated CLP group at 24 h after CLP injury in kidney (*P*=0.950) and spleen (*P*=0.861) (Figure 2A). Also, the ratio of SW/BW and KW/BW showed no significant difference in the kaempferol-treated CLP group compared with CLP group (*P*=0.686 and *P*=0.219). However, kaempferol treatment could increase the ratio of heart-to-body weight (HW/BW) at 24 h after CLP injury compared with sham or CLP alone. After 48 h time point in CLP injury, our data demonstrated that the ratio of SW/BW would be decreased (*P*=0.158) but HW/BW and KW/BW would be unchanged in kaempferol treatment (Figure 2A).

Furthermore, we performed H&E staining to identify the difference of the morphology of the kidney after CLP injury and found that the renal tubular became wrinkled and its arrangement was irregular at 24 h. Kaempferol treatment did not alleviate kidney damage at 24 h after CLP injury, and there was no difference between the kaempferol-treated CLP group and saline-treated CLP group at 48 h (Figure 2B). However, our qPCR data suggested that kaempferol would reduce significantly the expression of COX-2 besides IL-1β and TNF-α at 24 and 48 h after CLP injury (Figure 2C). These results illustrated that kaempferol would improve kidney injury by inhibiting inflammatory response in SAKI.

### Kaempferol inhibits the production of MCP-1, VCAM-1, and ICAM-1 in SAKI

**Figure 3:**
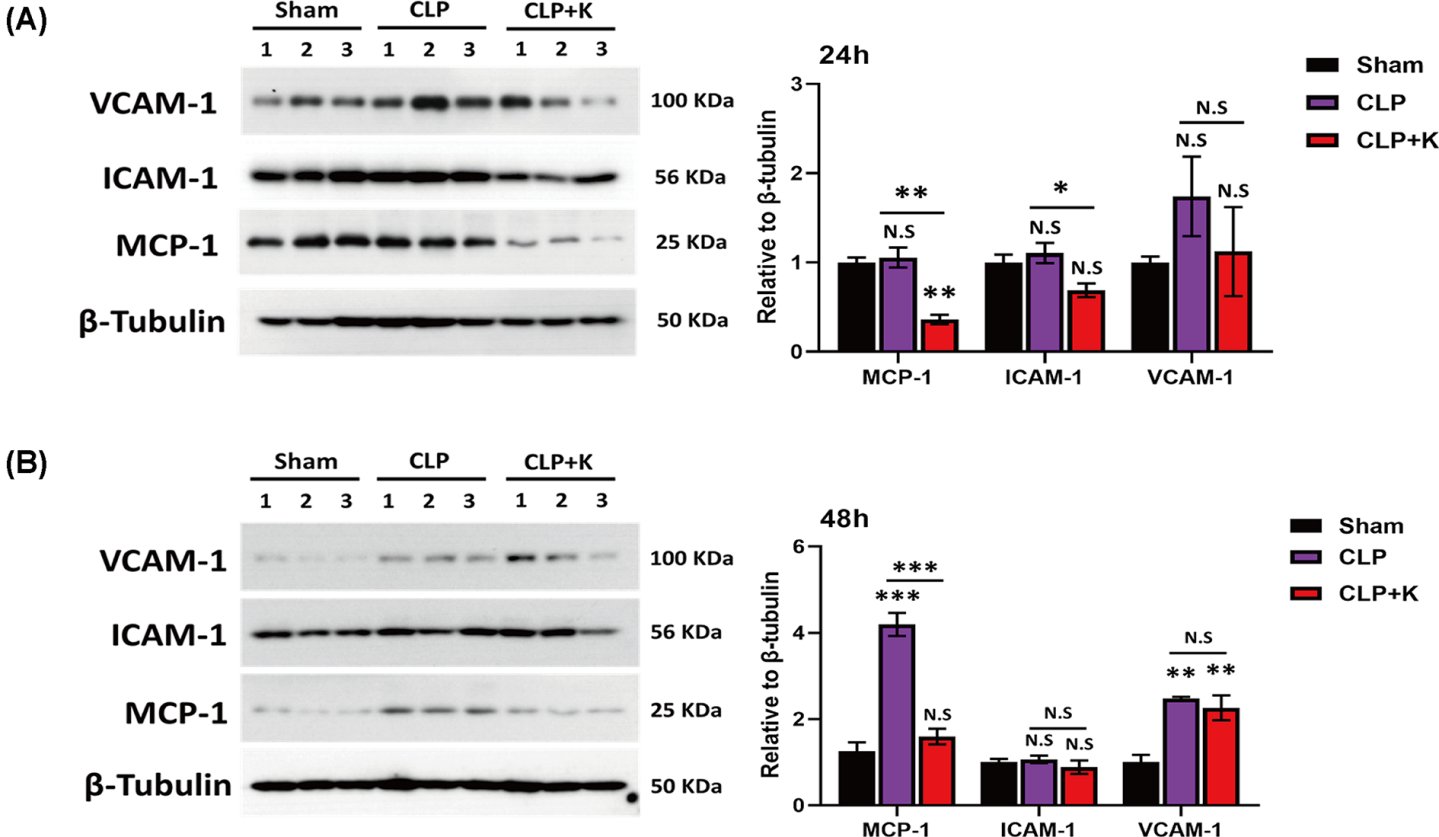
The production of MCP-1, VCAM-1, and ICAM-1 was significantly inhibited in Kaempferol treatment during sepsis (**A**) The protein expression level of MCP-1, VCAM-1, and ICAM-1 at 24 h; (**B**) protein expression level of MCP-1, VCAM-1, and ICAM-1 at 48 h in sham, saline-treated CLP mice, and kaempferol treated CLP mice (*n* = 3 per group, mean ± S.E.M, *P<0.05, **P<0.01, ***P<0.001).

Pro-inflammatory is a key mediator in the progress of sepsis, primarily leading to acute kidney injury. Santangelo et al demonstrated that different flavonoids decreased the production of pro-inflammatory markers, such as IL-6, TNF-α, IL-1β, and MCP-1 in RAW macrophages, Jurkat T-cells and peripheral blood mononuclear cells [35]. Also, flavonoids induced transcription factors such as (nuclear factor E2-related factor) Nrf2, an antioxidant responsive element (ARE), which regulated the expression of antioxidant proteins. Nrf2 suppressed the production of MCP-1 and VCAM-1 and thereby decreased monocyte adhesion and transmigration to endothelial cells in mice and rabbits [36]. Thus, we performed qPCR and Western blot to identify the production of pro-inflammatory genes (MCP-1, VCAM-1, and ICAM-1) after kaempferol treatment in CLP mouse model. The qPCR results showed that kaempferol inhibited significantly the mRNA level of ICAM-1 compared with saline-treated CLP group at 24 h, but there were no significant changes of MCP-1 and VCAM-1 in sepsis (Figure 2D). Also, the mRNA levels of MCP-1 (mean: 82.78 vs 65.38), ICAM-1 (mean: 11.45 vs 7.53) and VCAM-1 (mean: 5.13 vs 2.64) would reduce at 48 h time point after CLP injury in kaempferol treatment (Figure 2D). In addition, we further detected the protein levels of these pro-inflammatory genes by western blot in sepsis. Our data found that kaempferol reduced significantly the expression of MCP-1 and ICAM-1 at 24 h after CLP injury, and the protein level of VCAM-1 was decreased but not statistical difference (Figure 3A). We also found that the protein level of MCP-1 in the kaempferol-treated CLP group was significantly lower than that in the saline-treated CLP group at 48 h (Figure 3B). Therefore, our findings uncovered that kaempferol could inhibit MCP-1 expression to alleviate the inflammatory response of CLP injury in mice model.

### Kaempferol inhibits macrophage infiltration in SAKI

**Figure 4:**
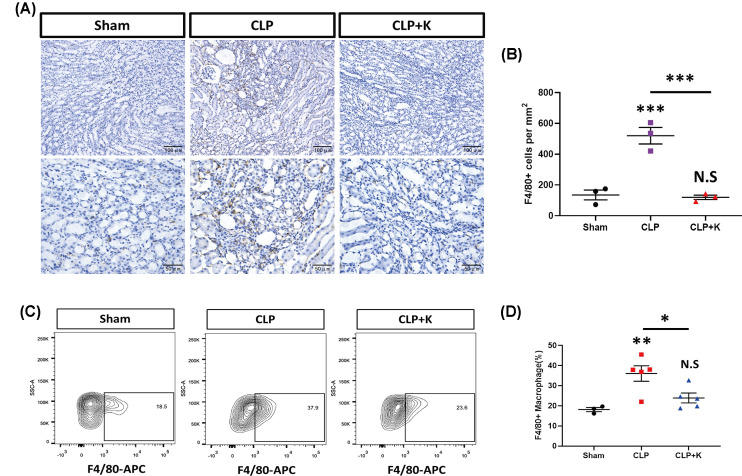
Kaempferol treatment reduced macrophage infiltration during sepsis (**A**) F4/80 IHC staining of kidney tissues and (**B**) quantification of F4/80 positive cells in sham, saline-treated CLP mice, and kaempferol treated CLP mice at 24 and 48 h. (**C**) flow cytometry analysis of F4/80 positive cells in kidney tissues and (**D**) quantification of F4/80 positive cells in sham, saline-treated CLP mice, and kaempferol treated CLP mice at 24 h (*n* = 3–5 per group, mean±S.E.M, *P<0.05, **P<0.01, ***P<0.001).

MCP-1/CCR-2 signaling was responsible for macrophage recruitment in tissue injury and inflammation. The inhibition of MCP-1/CCR-2 decreased macrophages infiltration and successfully alleviated the progression of diabetic nephropathy [37]. Thus, we asked if kaempferol would suppress macrophage recruitment by MCP-1 inhibition in SAKI. Our data suggested that CLP injury could promote macrophage infiltration in the kidney at 24 and 48 h (Figure 4A), which was consistent with a previous report [38]. More interestingly, kaempferol would suppress macrophage infiltration during sepsis, and the number of F4/80+ macrophages reduced significantly in the kaempferol-treated CLP group compared with saline-treated CLP group (Figure 4A,B). Our further FACS data also demonstrated that kaempferol inhibited significantly F4/80+ macrophage infiltration in kidney tissues after CLP injury (Figure 4C,D). These data demonstrated that kaempferol treatment could inhibit the infiltration of macrophages in mice, which contributed to alleviate SAKI.

## Discussion

The increased expression of PCT and C-RP has been detected during infection [39,40]. The serum level of PCT was lower in normal conditions, but that of PCT increased rapidly from 2 to 6 h after infection [41]. LPS activated NF-κB and STAT3, and then increased PCT expression [42]. Accumulating studies have confirmed that the serum level of CRP and PCT in septic patients increased significantly at 24 h [43–45]. Immune cells, such as white blood cells and lymphocytes, were responsible for the balance between the host immune response and microorganism infection during sepsis. Also, platelets plays an important role in the inflammatory response of sepsis, which participated in thrombosis and the formation of neutrophil extracellular traps (NETs) [46]. In this study, the serum level of PCT and C-RP up-regulated significantly in septic patients, which was consistent with previous studies. Also, the number of white blood cells increased considerably, but lymphocytes and platelets decreased remarkably in sepsis. These clinical data showed that inflammatory markers/cells were important mediators in the process of sepsis, leading to organ damage and dysfunction. Thus, the advanced treatments should be focused on how to regulate the balance between inflammatory response and anti-inflammatory response during the early stage of sepsis.

Several flavonoids are potential inhibitors of inflammatory response in macrophage, such as kaempferol, luteolin, genistein, epigallocatechin and wogonin, which decreased the expression of TNF-α and NF-κB in LPS treated macrophage [47,48]. In context of sepsis model, kaempferol plays an important role in anti-inflammatory response. Kaempferol would improve liver injury in sepsis rat model by selective COX-2 inhibition [23]. Bian et al reported that kaempferol reduced excessive production of TNF-α, IL-1β, IL-6, ICAM-1, and VCAM-1 in LPS group [24]. More interestingly, our study demonstrated that kaempferol could not only alleviate COX-2, but also inhibit the production of MCP-1, ICAM-1, and VCAM-1 at 24 h time point in CLP induced acute kidney injury.

Interestingly, kaempferol reduced the production of pro-inflammatory cytokines and subsequently inhibited monocyte adhesion and migration. Kaempferol inhibited NF-κB /Nrf2 activation, which contributed to alleviate renal injury in mouse model [49]. Also, Nrf2 decreased the expression of MCP-1 and VCAM-1 and consequently reduced monocyte adhesion and transmigration to endothelial cells, which reduced MAPK and p38 expression and alleviated the formation of atherosclerotic lesions in mice and rabbits [36]. Our findings indicated that kaempferol could reduce the expression of MCP-1 at 24 and 48 h time point, thereby inhibiting macrophage infiltration in septic group. In addition to its role in MCP-1/CCR-2 signaling pathway of macrophage, the further studies would be investigated whether kaempferol regulates Nrf2/MCP-1 pathway to reduce macrophages infiltration in septic mouse model.

Taken together, our results showed that kaempferol contributed to suppress the inflammatory response by inhibiting inflammatory markers, especially MCP-1 expression, and consequently reduce macrophage infiltration in CLP-induced acute kidney injury, which alleviated organ damage and dysfunction during sepsis.

## Supplementary Material

Supplementary Table S1

## Data Availability

All data included in this study are available upon request by contact with the corresponding author

## Abbreviations

ARE: antioxidant responsive element
CLP: cecal ligation and puncture
COX-2: Cyclooxygenase-2
C-RP: C-reactive protein
ICAM-1: Intercellular cell adhesion molecule-1
IL-1β: Interleukin-1β
IL-6: Interleukin-6
MCP-1: monocyte chemoattractant protein-1
NET: neutrophil extracellular trap
PCT: procalcitonin
PVDF: polyvinylidene fluoride
SAKI: sepsis-associated acute kidney injury
TNF-α: tumor necrosis factor-α
VCAM-1: vascular cell adhesion molecule-1
