# Supplementary material for: Kaempferol improves acute kidney injury via inhibition of macrophage infiltration in septic mice
Source: Biosci Rep. 2023 Jul 25;43(7):BSR20230873. doi: 10.1042/BSR20230873 (PMC10372469; doi:10.1042/BSR20230873)
Supplement: Supplementary Table S1 [file BSR-2023-0873_supp.pdf]

Table_S1. Listing of primers and primer sequences for real-time PCR

No.	Gene name	Primer sequence (5′-3′)
1	TNF-α	ATTATGGCTCAGGGTCCAAC
		GACAGAGGCAACCTGACCAC
2	IL-1β	GCTGCTTCCAAACCTTTGACC
		AGCCACAATGAGTGATACTGCC
3	COX-2	TGCTGTACAAGCAGTGGCAA
		GCAGCCATTTCCTTCTCTCC
4	ICAM-1	CTCACTTGCAGCACTACGG
		TTCATTCTCAAAACTGACAGGC
5	VCAM-1	GCCACCCTCACCTTAATTGCT
		GCACACGTCAGAACAACCGAA
6	MCP-1	TTAAAAACCTGGATCGGAACCAA
		GCATTAGCTTCAGATTTACGGGT
7	GAPDH	GGCATTGCTCTCAATGACAA
		TGTGAGGGAGATGCTCAGTG
